# Quantitative ophthalmic posterior segment optical coherence tomography angiography and neurologic conditions: a review

**DOI:** 10.3389/fneur.2026.1746695

**Published:** 2026-01-23

**Authors:** Michael Drakopoulos, Clayton E. Lyons, Hayden Sikora, Sabra Abbott, Nicholas Volpe, Rukhsana G. Mirza

**Affiliations:** Department of Ophthalmology, Northwestern University Feinberg School of Medicine, Chicago, IL, United States

**Keywords:** choriocapillaris, deep capillary plexus, intermediate capillary plexus, oculomics, optic nerve head, optical coherence tomography angiography, radial peripapillary capillary plexus, superficial capillary plexus

## Abstract

**Objectives:**

To investigate the potential of quantitative ophthalmic posterior segment optical coherence tomography angiography (OCTA) imaging metrics to serve as biomarkers for systemic involvement in five neurologic diseases (multiple sclerosis, neuromyelitis optica spectrum disorder, myelin oligodendrocyte glycoprotein antibody-associated disease, Alzheimer disease, and Parkinson disease) by reviewing the reported correlations between such OCTA metrics and clinically relevant features of systemic involvement in these diseases.

**Methods:**

This article is a literature review of the PubMed database for articles reporting OCTA metrics in any of the included neurologic diseases. Articles correlating quantitative retinal, optic nerve head, or choriocapillaris OCTA metrics to clinically relevant features of systemic involvement, specifically serum, cerebrospinal fluid (CSF), or other established biomarkers; genotype; systemic symptom and severity scores; stage; non-ocular organ involvement; brain or other non-ocular imaging findings; and systemic medication use were included.

**Results:**

OCTA parameters have been significantly correlated to established biomarkers, severity scores, non-ocular organ involvement and imaging findings, and systemic medication use in multiple sclerosis. OCTA parameters have been significantly correlated to established biomarkers, severity scores, and non-ocular organ involvement and imaging findings in neuromyelitis optica spectrum disorder. OCTA parameters have been significantly correlated to severity scores in myelin oligodendrocyte glycoprotein antibody-associated disease. OCTA parameters have been significantly correlated to established biomarkers, genotype, severity scores, disease stage, and non-ocular organ involvement and imaging findings in Alzheimer disease. OCTA parameters have been significantly correlated to severity scores, disease stage, and non-ocular organ involvement in Parkinson disease.

**Conclusion:**

Our findings suggest that ophthalmic posterior segment OCTA might improve our understanding of the pathophysiology of systemic neurologic conditions, including those that do not traditionally affect the eye, and might identify biomarkers useful in the diagnosis, prognosis, and management of these conditions, justifying further investigation.

## 1 Introduction

The human retina and brain share a common diencephalic embryologic origin (1). Both organs exhibit extremely high oxygen demand per unit volume compared to the rest of the body and have unique autoregulatory mechanisms to maintain a relatively constant blood flow in response to local neuronal activity (2). Due to these striking similarities, and likely shared neurovascular, inflammatory, and neurodegenerative mechanisms, changes in the retinal blood vessels may reflect alterations in the vasculature of the brain.

**Figure 1:**
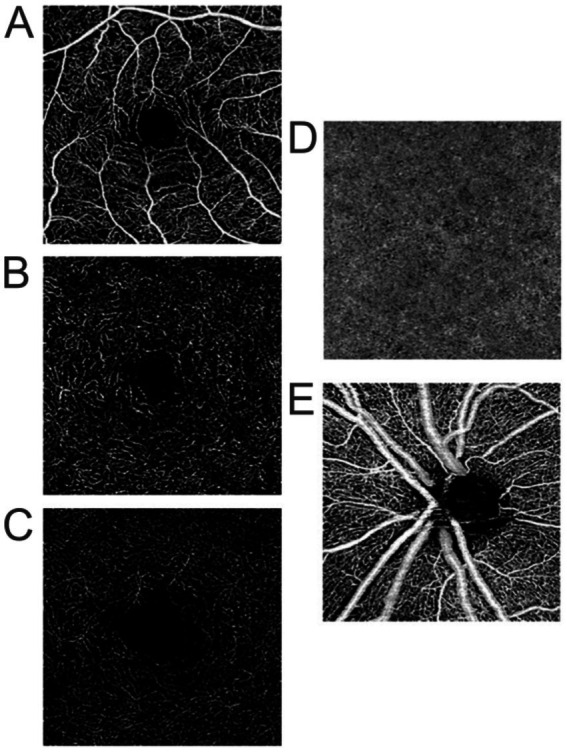
Optical coherence tomography angiography en face 3 mm × 3 mm images of the **(A)** superficial capillary plexus (SCP), **(B)** intermediate capillary plexus (ICP), **(C)** deep capillary plexus (DCP), **(D)** choriocapillaris (CC), **(E)** radial peripapillary capillary plexus (RPCP).

Importantly, the retina’s anatomic location at the posterior pole of the eye provides an opportunity to evaluate central nervous system (CNS) vasculature through direct visualization. Optical coherence tomography angiography (OCTA) was developed as a tool to non-invasively assess the vasculature in tissues such as the skin and retina. OCTA provides in-vivo, depth-resolved visualization of all layers of the retinal microvasculature including the superficial, intermediate, and deep capillary plexuses (SCP, ICP, and DCP, respectively), and the radial peripapillary capillary plexus (RPCP), as well as of the choriocapillaris (CC) (1) (Figure 1). Through image processing, OCTA provides quantitative metrics such as vessel area density (VAD), vessel length density (VLD), foveal avascular zone (FAZ) area, and FAZ circularity. All quantitative OCTA metrics included in this review are named and defined in Table 1.

**Table 1:** Names, abbreviations, and definitions of the quantitative ophthalmic posterior segment optical coherence tomography angiography metrics relevant to this review.

Metric name (abbreviation)	Definition
Vessel area density (VAD)	The area of retina covered by perfused blood vessels divided by the total area of interest of the en face retinal image; generally measured after binarization
Vessel length density (VLD)	The total length of 1-pixel-wide, skeletonized perfused blood vessels of the en face retinal image divided by the total retinal area of interest
Volumetric vessel density (VVD)	Fractal dimension of an en face retinal image following skeletonization divided by the volume of tissue in the region of interest
Foveal avascular zone area (FAZ area)	Total area of the foveal avascular zone
Foveal avascular zone circularity index (FAZ circularity index)	A metric calculated as 4πA/P^2^ where A = FAZ area and P = FAZ perimeter. Values close to 0 indicate an irregular shape; values close to 1 indicate a circular shape
Fractal dimension (FD)	A value calculated automatically by a computer program that describes the dimension of a fractal, which is a geometric pattern that branching blood vessels mimic
Periarteriolar capillary free zone width	The average width of the capillary-free zone surrounding the ophthalmic posterior segment arterioles (larger distances indicate potential ischemia)
Peripapillary flow index	The area of perfused blood vessels divided by the area of interest and weighted by the intensity of the local flow signal in the peripapillary region
Perivenular capillary free zone width	The average width of the capillary-free zone surrounding the ophthalmic posterior segment venules (larger distances indicate potential ischemia)
Optic nerve head flow index	The area of perfused blood vessels within the optic disc divided by area of interest and weighted by the intensity of the local flow signal

These parameters, which have been applied to a variety of ocular pathologies including age-related macular degeneration (AMD), macular telangiectasia, diabetic retinopathy and glaucoma among others (3) and have recently been applied in the emerging field of oculomics, which seeks to utilize ophthalmic imaging to identify ocular biomarkers for systemic diseases. Changes in the retina identified by OCTA have been correlated with clinically relevant features of various systemic conditions (4), including rheumatologic (5), vascular (6), and neurologic (4) conditions. In this review, we summarize the current OCTA literature on systemic correlations in several of the most common neurologic diseases including multiple sclerosis (MS), neuromyelitis optica spectrum disorder (NMOSD), myelin oligodendrocyte glycoprotein antibody-associated disease (MOGAD), Alzheimer disease (AD), and Parkinson disease (PD). Prior reviews have discussed one or more of the included conditions, though no review has integrated all five selected diseases and exclusively focused on systemic correlates (1–3, 7–46). Further, by focusing on systemic correlations, we are able to identify areas in which OCTA may be capable of serving as a surrogate for various aspects of disease such as disability, cognitive function, symptom burden, and longitudinal symptom progression as well as traditional markers of disease, such as neuroimaging and serologic features of diseases, that are either more invasive or more costly to obtain. Eye-specific correlations between OCTA and other ocular imaging modalities such as conventional OCT are not discussed, as they have been presented elsewhere (47–50). We hypothesized that correlations between OCTA metrics and systemic markers of the included neurologic diseases would be identified, demonstrating the potential of OCTA metrics as possible surrogates for systemic activity in these neurologic diseases.

## 2 Methods

This review was a literature review of correlational studies. We performed a literature review of the PubMed database to identify studies reporting correlations between quantitative ophthalmic posterior segment OCTA metrics and non-ocular markers of clinical disease in MS, NMOSD, MOGAD, AD, and PD. The following non-ocular markers were considered: serum, cerebrospinal fluid (CSF), or other established biomarkers; genotype; systemic symptom and severity scores; stage; non-ocular organ involvement; brain or other non-ocular imaging findings; and systemic medication use. The diseases of interest were chosen based on the expert opinion of a medical retina specialist (RGM), due to these conditions being frequently reported and to include conditions both with and without frequent eye involvement. While MS, NMOSD, MOGAD, have classic eye manifestations, especially optic neuritis, AD and PD have not been reported to have classic eye findings. Neurologic diseases both with and without classic eye manifestations were included to examine the ability of OCTA to identify ophthalmic involvement and its systemic correlates even in neurologic conditions where such correlations were less likely to be found. Search queries for each disease were entered into the PubMed database on August 3rd, 2024, by a single investigator. Searches were limited to works published in English evaluating human subjects after January 1st, 2000, as OCTA was not used clinically prior to 2000. A sample search query, for Alzheimer disease, and the key words for each search query are presented in Table 2.

**Table 2:** Sample PubMed search query (for Alzheimer disease) and key words used in PubMed database search.

Sample search query (Alzheimer disease)
((“Optical coherence tomography angiography”) OR (“OCT angiography”) OR (“OCT-angiography”) OR (“OCT-A”) OR (“OCTA”)) AND ((“Alzheimer disease”) OR (“Alzheimer’s disease”) OR (“Alzheimers disease”) OR (“Alzheimers”) OR (“Alzheimer”) OR (“AD”))

Disease included	Key words used in search query
Alzheimer disease	Alzheimer disease, Alzheimer’s disease, Alzheimers disease, Alzheimers, Alzheimer, AD
Multiple sclerosis	Multiple sclerosis, MS
Myelin oligodendrocyte glycoprotein antibody-associated disease	Myelin oligodendrocyte glycoprotein antibody-associated disease, myelin oligodendrocyte glycoprotein antibody associated disease, myelin oligodendrocyte glycoprotein antibody-associated disorder, myelin oligodendrocyte glycoprotein antibody associated disorder, MOG antibody-associated disease, MOG antibody-associated disorder, MOG antibody disease, MOG antibody disorder, MOGAD
Neuromyelitis optica spectrum disorder	Neuromyelitis optica spectrum disorder, neuromyelitis optica spectrum disorders, neuromyelitis optica, Devic disease, NMOSD, NMO
Parkinson disease	Parkinson disease, Parkinson’s disease, Parkinsons disease, Parkinsons, Parkinson, PD

The identified articles were deduplicated. Abstracts were screened by a single grader. Articles with abstracts describing the following study characteristics were excluded: case reports, case series with fewer than ten subjects with the neurologic disease of interest, clinical-trial protocols without data or other protocols without data, editorial publications not presenting new data, publications unrelated to OCTA, publications failing to discuss ophthalmic-posterior-segment OCTA, publications correlating ophthalmic OCTA findings only to diseases other than MS, NMOSD, MOGAD, AD, and PD, and any remaining non-English or non-human-subjects articles. The texts of the remaining articles were further screened by single-grader review to exclude those with no quantitative OCTA metrics, those irreversibly combining OCTA metrics with laboratory or imaging metrics, and those failing to correlate quantitative posterior segment OCTA metrics in the included neurologic diseases to the previously noted clinically relevant non-ocular metrics or markers of those diseases. Reference lists of the included articles were also reviewed to identify additional relevant studies.

## 3 Results

**Figure 2:**
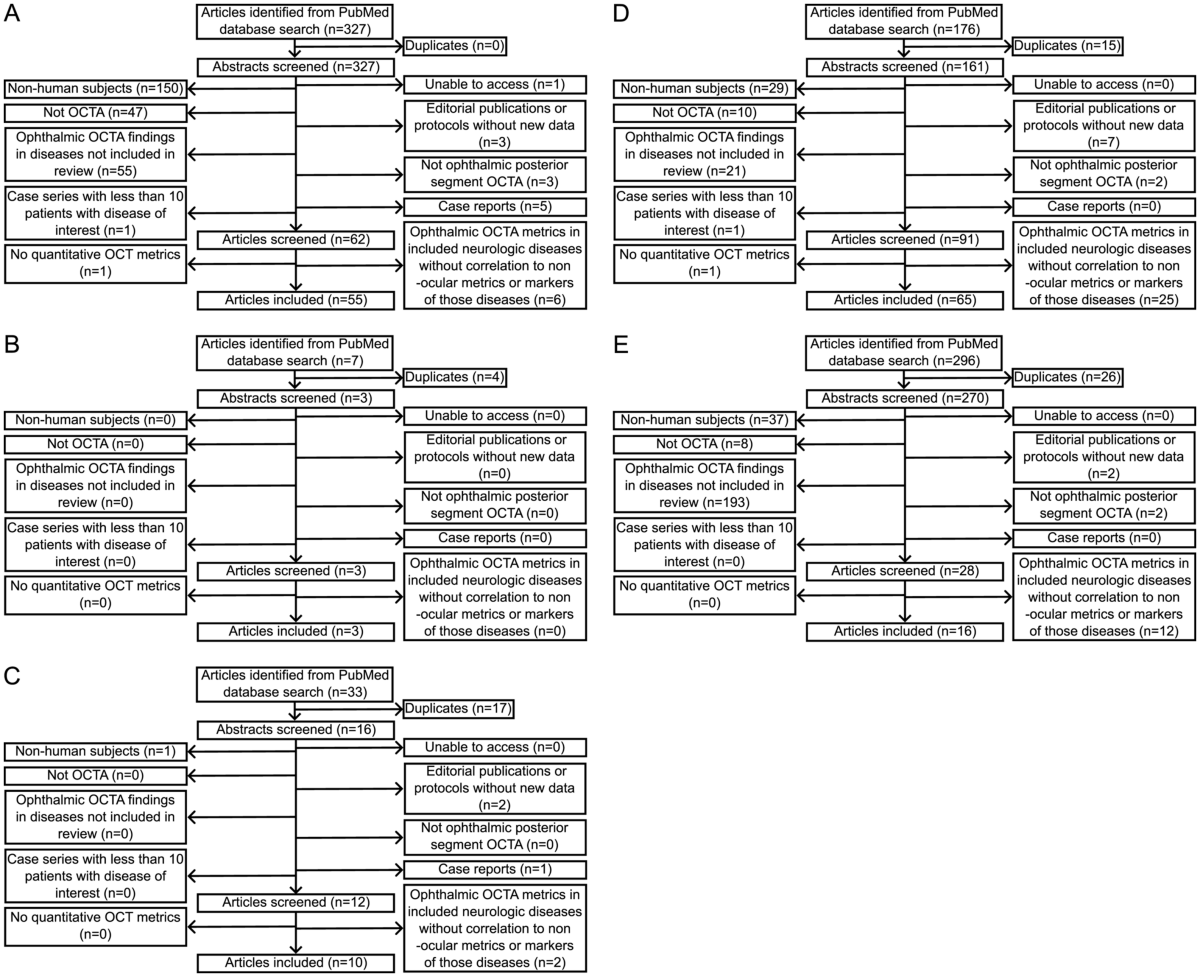
Flowcharts of numbers of articles included and excluded after PubMed database searches for each of **(A)** multiple sclerosis, **(B)** myelin oligodendrocyte glycoprotein antibody-associated disease, **(C)** neuromyelitis optica spectrum disorder, **(D)** Alzheimer disease, and **(E)** Parkinson disease with the queries indicated in Table 2.

778 different articles were identified based on search queries. 588 articles were excluded following abstract review, 1 article was excluded due to lack of access, and 43 articles were excluded after article review. In total, 147 articles met inclusion criteria. A flowchart outlining the inclusion and exclusion of studies for each disease is provided in Figure 2.

### 3.1 Multiple sclerosis

Multiple sclerosis (MS) is a neuro-inflammatory autoimmune disease that results in axonal demyelination in the CNS (1). MS represents the most common chronic inflammatory disorder of the CNS, and its prevalence has been increasing over the last several years, particularly in young individuals living in developed countries (2, 11). Approximately half of all MS patients will experience optic neuritis (ON) during their disease course (1). In fact, optic nerve degeneration and axonal loss has been observed in 90% of MS patients post-mortem, regardless of ON history (8). While it is broadly accepted that inflammation is a prominent contributor to the pathophysiology of MS, there is emerging evidence that metabolic and vascular disturbances may also occur and result in neuronal damage (1). OCTA may be capable of capturing these vascular changes, and we review its potential clinical utility in MS below. Notably, it remains unclear if OCTA metrics change before or after retinal thinning noted on optical coherence tomography images, and it is possible that inflammation leading to thinning causes pruning of the ophthalmic microvasculature visible on OCTA. Alternatively, primary damage to blood vessels might lead to tissue thinning.

#### 3.1.1 Correlation with disability and EDSS score

Several studies have examined the correlation between OCTA parameters and disability in MS, as quantified by the Expanded Disability Status Scale (EDSS) with higher scores representing greater disability. Cross-sectional studies have occasionally found that macular, peripapillary, and CC VAD as well as CC VLD are inversely correlated with EDSS (51–54), while others have not identified a significant relationship between the two variables (48, 55–60). Interestingly, one analysis found an inverse correlation between a reduction in SCP vessels with a diameter <10 μm and EDSS score, while this was not observed with medium-sized vessels (diameter 10–20 μm) or large-sized vessels (diameter >20 μm) (61). This suggests that VLD, which more heavily weights capillaries compared to VAD, may exhibit a stronger relationship with EDSS. Most studies to date have focused on VAD warranting further study of the relationship between VLD and EDSS. Interestingly, the only study that evaluated macular VVD, which incorporates retinal tissue volume into the VLD metric, identified a positive rather than negative correlation between entire retina and SCP VVD and EDSS (62).

Longitudinally, reductions in SCP and DCP VAD have been correlated with increases in EDSS (63, 64). Reductions in SCP and DCP VAD have also been shown to be capable of predicting worsening disability in MS over the course of nearly 2 years of follow-up (65). Broadly, then, reduced retinal perfusion may be able to serve as a surrogate for worsening disability in this condition. In addition, those with an EDSS ≤2 saw a significant reduction in DCP VLD from baseline after 2 years of follow-up compared to those with EDSS >2 at baseline (66), which suggests that vascular changes in MS may occur even with little overall disease burden prior to reaching a plateau with disease progression. This finding also suggests that changes in OCTA metrics might occur earlier in disease, when EDSS might be lower. Notably, OCTA parameters do not appear to exhibit a relationship with relapse rate in MS (56, 59, 67, 68), although increased CC VAD has been associated with ongoing inflammatory disease activity (69). Notable limitations of these studies include excluding patients with relapses within the last month who may have more severe disease and failing to describe in detail the specific type of DMT the participants received (56, 59, 68). The latter limitation is particularly important, as newer DMT significantly reduces relapse rates compared to older therapies (70).

#### 3.1.2 OCTA differences early in the disease course

Compared to patients following an initial demyelinating event (IDE), patients diagnosed with MS have decreased RPCP VAD (58). A limitation of this study was that time from IDE was not reported, making it unclear how much time had passed between the IDE and OCTA acquisition. A different study, however, examined patients 2 years following an IDE, which revealed reductions in SCP, DCP, and RPCP VAD from baseline whereas healthy controls did not (59).

#### 3.1.3 Correlation with neuroimaging

For those with a diagnosis of MS, the retinal microvasculature may also provide insight into changes in the brain. Reductions in SCP VAD have been correlated with both gray and white matter atrophy on magnetic resonance imaging (MRI), while DCP VAD loss has been associated with gray matter atrophy (64). Higher T2-weighted lesion load has also been correlated with reduced peripapillary VAD (52). Interestingly, the associations of OCTA with MRI signs of disease activity have not been observed early in the disease course, as changes on OCTA did not exhibit a relationship with radiological disease activity in a 2-year follow-up period after an IDE (59). This lack of correlation may be due to the retina more readily showing changes on OCTA due to its microscopic and quantitative nature compared to the more macroscopic changes that need to take place to be detectable on MRI. An alternate explanation is that the retina independently shows changes earlier in the disease course. Regardless, these findings suggest both that OCTA metrics might correlate closely with MRI findings and that ophthalmic microvascular changes might be used both to monitor MS and provide insight into its pathogenesis.

#### 3.1.4 Correlation with serum and CSF biomarkers

Negative correlations have been identified between macular SCP and DCP VAD and intrathecal concentration of activated B cells, a well-established marker of MS disease activity (65). SCP and DCP VAD have also shown significant negative correlations with CSF levels of various proinflammatory cytokines (65). Though few studies have explored these relationships, the initial data suggest that OCTA may reflect alterations in known biomarkers of MS disease activity.

#### 3.1.5 Measuring response to disease-modifying therapy

Few studies have explored the relationship between OCTA parameters and exposure to disease modifying therapy (DMT). MS patients receiving fingolimod specifically for greater than 6 months have exhibited an increased FAZ area compared to those receiving therapy for less than 6 months as well as healthy controls (71). Another study found that DMT exposure did not affect the rate of change of OCTA parameters over nearly 2 years of follow-up (64). A major limitation of this study, however, was the cohort received a wide variety of DMT rather than a single agent.

#### 3.1.6 Differentiating MS from other autoimmune conditions

OCTA has also shown promise in differentiating MS from other autoimmune diseases that can affect the CNS. Patients with Sjogren syndrome, a potential MS mimic, exhibit a reduction in DCP VAD compared to healthy controls that was not observed in MS patients, though both conditions showed a reduction in SCP VAD (72). The loss of vessels in the SCP in MS patients is thought be secondary to retinal thinning reducing metabolic load resulting in decreased perfusion of retinal capillary beds, while the additional reduction in DCP VAD observed in Sjogren syndrome patients is theorized to be due to a primary vascular process, as Sjogren’s has been associated with retinal vasculitis (72). OCTA has also revealed a unique reduction in foveal SCP VAD and VLD as well as DCP VLD in those with rheumatologic disease with CNS involvement compared to MS patients (73). Of note, the study group of those with rheumatologic disease with CNS involvement included a variety of rheumatologic conditions including systemic lupus erythematosus, undifferentiated connective tissue disease, Sjogren syndrome, rheumatoid arthritis, neurosarcoidosis, juvenile idiopathic arthritis, and CNS vasculitis making it difficult to draw definitive conclusions from the study (73). Only one identified study compared OCTA parameters in the various MS phenotypes (relapsing remitting MS, primary progressive MS, and secondary progressive MS), and no significant differences were identified (56). These studies support the idea that OCTA may be able to aid clinicians in the differential diagnosis of conditions with similar clinical presentations. It is important to note, however, that the discussed literature focuses on differentiating those patients with established diagnoses; additional work must be done to establish OCTA as a clinically useful tool in differentiating patients with disease from disease suspects without disease. A discussion of the literature on OCTA’s ability to distinguish MS from NMOSD is included in the NMOSD section of the review.

#### 3.1.7 Challenges, considerations, and conclusions

An important caveat to all OCTA studies in MS patients is that MS patients have been found more likely to have imaging artifacts that result in an artificially low VAD (74). This increase in motion artifacts specifically is thought to be due to both past and present ON contributing to poor fixation during image acquisition (9). This finding underscores the importance of appropriately trained staff following established image acquisition protocols as well as the development of normative OCTA data for those with and without MS to ensure the validity of future studies.

In conclusion, several relationships have been observed between OCTA parameters and various markers of disease in MS. Broadly, retinal microvascular rarefication has been observed following IDEs, exhibited an inverse relationship with EDSS scores and markers of disease activity, and been correlated with MRI signs of brain atrophy. It is important to note that retinal thinning has been widely observed in MS eyes, with a greater reduction in retinal thickness observed in ON eyes, making the observed retinal vessel loss a potential sequela of reduced retinal oxygen demand subsequent to tissue loss following inflammation (75). However, it is possible the observed capillary rarefication represents an independent vascular component of MS pathology (75). Establishing temporality of these changes requires further longitudinal study and may provide critical insight into the complex pathophysiology of MS.

### 3.2 Neuromyelitis optica spectrum disorder

Neuromyelitis optica spectrum disorder (NMOSD) is another inflammatory, autoimmune condition of the CNS that classically presents with transverse myelitis and acute optic neuritis (76). Due to their significant overlapping features, individuals with NMOSD were previously diagnosed as having MS, but the discovery of the IgG autoantibody to aquaporin 4 (AQP4) has led to its classification as an independent disease (77). Approximately 70–90% of NMOSD patients are seropositive for AQP4-antibodies, though patients can be seronegative (77). The disease is quite rare, with incidence estimated to be less than one per million per year in the general population, and prevalence of 1/100,000 among those identifying as white, 3.5/100,000 among those identifying as east Asian, and 10/100,000 among those identifying as black (78).

#### 3.2.1 Correlation with disability and EDSS

As in MS, disability in NMOSD is often quantified by EDSS, which has shown several correlations with OCTA parameters in NMOSD patients. Increases in EDSS have been correlated with decreased SCP fractal dimension (FD) and increased FAZ area (79, 80). Studies investigating the relationship between EDSS and VAD have yielded variable results. In the SCP, increased EDSS has generally been associated with decreases in SCP VAD (49, 77, 81), though one study did not find an association (82). A negative correlation has also been observed between EDSS and ICP VAD (49) as well as EDSS and RPCP VAD (77, 81), though again one study did not identify an association between EDSS and ICP VAD (82). The relationship between EDSS and DCP VAD is less clear with some studies not identifying an association (81, 82), another identifying a positive correlation (49), and yet another identifying a negative correlation (77).

#### 3.2.2 Correlation with neuroimaging

Fewer studies have explored the relationship between OCTA and CNS imaging findings in NMOSD than in MS. This may be due to NMOSD more commonly affecting the optic nerve and spinal cord rather than brain; however, studies have found that a positive correlation exists between RPCP VAD and the functional connectivity of the right lingual gyrus, bilateral calcarine gyrus, left thalamus, and right superior parietal gyrus in NMOSD patients (83, 84), while a negative correlation exists between RPCP VAD and the functional connectivity of the right fusiform gyrus, left orbital portion of superior frontal orbital gyrus, left anterior cingulum, and paracingulate gyri (84). With further study, OCTA metrics may prove capable of providing information on the functionality of various brain regions in NMOSD.

#### 3.2.3 Correlation with serum markers

OCTA parameters have also shown significant correlations with serum biomarkers of disease activity in NMOSD. For example, increased FAZ area and decreased SCP VAD have been correlated with increased serum glial fibrillary acidic protein (GFAP) levels in NMOSD patients without a history of ON (80). GFAP is an intermediate filament protein found in the cytoskeleton of astrocytes and only released after cell death or injury, broadly serving as a surrogate of CNS disease activity (85). Studies investigating OCTA’s relationship with serum neurofilament light chain (sNfL), another biomarker of neuronal damage, have been less conclusive. One study found a significant positive correlation between sNfL and SCP VAD in NMOSD patients without a history of ON (67), while another study did not find a meaningful relationship between the two variables (80). Nonetheless, these results suggest that different OCTA parameters may be able to capture collective CNS damage in NMOSD with further study.

#### 3.2.4 Differentiating NMOSD from MS

RPCP VAD has shown the most promise in aiding clinicians in differentiating NMOSD from MS. When comparing patients with a history of ON in both conditions, those with NMOSD have been found to have lower RPCP VAD than MS patients (86–89). This relationship has been particularly notable in the superior and inferior sectors (87). This may be a result of those with NMOSD typically having more severe ON than those with MS. Study of other OCTA parameter differences between the two conditions has been more mixed. Inconsistent decreases in SCP and DCP VAD have been observed in NMOSD compared to MS patients (80, 86–88, 90). Analyzing FAZ area differences between those with NMOSD and MS has yielded conflicting results, with one study identifying an increase in the FAZ area in NMOSD with no history of ON (80), while another study found decreased FAZ area and perimeter in NMOSD patients without a history of ON (88). Importantly, adding temporal-inner quadrant VLD and temporal-inner, nasal-inferior, and nasal-outer quadrant VAD to structural OCT data improved diagnostic discrimination between NMOSD and MS from AUC = 0.768 with OCT alone to AUC = 0.833 with OCTA added (90), demonstrating additional value of OCTA in differentiating these conditions.

#### 3.2.5 Conclusion

Broadly, RPCP VAD has demonstrated the most consistent associations with features of NMOSD with the metric exhibiting inverse correlations with EDSS, significant relationships with functional connectivity of the brain, and the most promise in differentiating NMOSD from MS. Most NMOSD cases have localized pathology to the optic nerve and spinal cord whereas MS frequently involves the development of lesions throughout the CNS. This may be due to the localization of AQP4 channels, antibodies to which drive the pathogenesis of NMOSD. Further, it has been shown that NMOSD patients tend to have more frequent recurrences of ON with bilateral ON often observed at first ON attack, whereas MS patients typically have unilateral ON, resulting in NMOSD patients exhibiting poorer long-term visual outcomes (91). With the optic nerve more frequently and severely affected in NMOSD, it follows that the microvasculature surrounding the optic nerve would be more adversely affected in NMOSD. Further, since the microvasculature is reduced in both MS and NMOSD with ON, and was seen to correlate with optic neuritis attacks in NMOSD, it is possible these vessel metrics might serve as an indicatory of poor prognosis of visual function in these conditions.

Quickly differentiating NMOSD from MS is particularly critical, as some immunomodulatory MS treatments may exacerbate NMOSD (92). Further, while NMOSD is significantly less responsive to steroids than MS, retrospective studies suggest that initiating plasma exchange in NMOSD patients within 5 days of optic neuritis onset is associated with an increased probability of complete symptom resolution and better preservation of vision (92). With further study, OCTA may be able to aid in the rapid diagnosis of NMOSD subsequently improving the often-poor visual outcomes associated with the condition.

### 3.3 Myelin oligodendrocyte glycoprotein antibody-associated disease

Myelin oligodendrocyte glycoprotein antibody-associated disease (MOGAD) is yet another inflammatory, autoimmune condition that can present quite similarly to NMOSD, with optic neuritis being the most frequently observed symptom (93). Additional clinical manifestations include reduced vision, transverse myelitis, seizures, and encephalitis (49). The disease is mediated by antibodies against myelin oligodendrocyte glycoprotein, which is expressed in myelin sheaths throughout the CNS (49). Currently, diagnosis is confirmed through antibody testing, which is not commonly available (49). OCTA once again shows promise in bridging this diagnostic gap.

#### 3.3.1 Correlations to systemic metrics in MOGAD

Significantly fewer studies have examined OCTA metrics in MOGAD compared to MS and NMOSD, with most published works focusing on differences between MOGAD and NMOSD cohorts. In those with a history of ON, MOGAD individuals have exhibited decreased DCP VAD compared to those with NMOSD eyes (50), though a second study found no significant differences (49). This second study did, however, identify a reduction in the SCP VAD in MOGAD compared to NMOSD irrespective of ON history. MOGAD patients have also been observed to have a reduced FAZ area relative to NMOSD eyes (49). Collectively, these results suggest that OCTA may be useful in the differentiation of these clinically similar conditions with further study. Of note, SCP VAD has been inversely correlated with EDSS scores in MOGAD patients, suggesting OCTA may be able to serve as surrogate for disability in this condition as well (49).

#### 3.3.2 Conclusion

Although OCTA data in MOGAD are relatively sparse, initial studies suggest that macular VAD values are reduced in MOGAD compared to NMOSD. It is thought that this may be due to MOG antibodies causing more severe vascular damage, as a case of MOGAD initially presented as CNS vasculitis (50). Further study of the retinal microvascular changes in both entities is needed in order to draw definitive conclusions, however.

### 3.4 OCTA differences in MS, NMOSD, and MOGAD based on prior history of optic neuritis

Optic neuritis results from inflammation of the optic nerve (92). This inflammation can be the result of autoimmunity, infection, paraneoplastic processes, and demyelination (92). With a wide variety of potential causes, long-term outcomes vary considerably due to the underlying cause of optic neuritis. Traditional OCT has been used to identify initial thickening of the retinal nerve fiber layer (RNFL) in acute optic neuritis followed by RNFL and macular thinning with resolution of optic neuritis (92), with the finding of thinning after optic neuritis in MS first demonstrated on OCT imaging in 2006 (94). We explore the OCTA manifestations between those with and without ON in MS, NMOSD, and MOGAD below.

In MS, these differences have primarily been identified in the RPCP and SCP. Multiple groups have identified reduced RPCP VAD in those with a history of optic neuritis (MSON+) compared to those without (MSON-) (89, 95, 96). A similar reduction in MSON+ individuals has been observed in peripapillary flow index and optic nerve head flow index (96–99). Changes in the macula have also been reported, with MSON+ patients broadly showing decreased peripapillary and macular SCP VAD (99–101). Interestingly, one study identified significant variation in the difference between DCP and SCP VAD (DCP minus SCP), with MSON+ eyes showing a greater difference in the VAD of the two plexuses compared to non-ON eyes (102). It has been suggested that these alterations are specific to the eye affected by ON, as MSON+ patients have shown significantly greater inter-eye differences in SCP VAD at 1–3 years and >3 years following an ON episode, with the eye with ON having lower VAD (101). VVD of the full retinal network has also been shown to be greater in MSON+ eyes (103), likely due to retinal thinning. The choroidal vasculature has exhibited changes as well, with a reduced choroidal VAD in MSON+ (99, 104). Collectively, these studies suggest that optic neuritis may induce alterations in the ocular vasculature, though differences between MSON+ and MSON- cohorts have not been observed in all studies (54, 105). One study sought to establish a timeline of these vascular changes following an acute ON attack and found that reduction in SCP VAD evolves quite early and reaches a plateau 90–180 days following an attack (60). The group also found an increase in FAZ area within 180 days of an ON episode (60). Thus, OCTA may be particularly useful in identifying previously missed ON, triggering further work-up and potentially earlier diagnosis of MS in such individuals. Further, the recent investigations into the timeline of OCTA changes following an ON attack may prove useful in establishing temporality of attacks.

NMOSD patients with a history of optic neuritis (NMOSDON+) have been shown to have reduced RPCP VAD and SCP VAD compared to those with no history of optic neuritis (89, 106). As in MS, one study found significant variation in the difference between DCP and SCP VAD (DCP-SCP, with all DCP measures greater than SCP measures), with NMOSDON+ vasculature showing a greater difference between the VAD of the two plexuses compared to non-ON eyes (102). In NMOSD specifically, reductions in SCP and DCP VAD have exhibited a positive correlation with frequency of optic neuritis attacks (107). Significant differences between NMOSDON+, NMOSDON-, MSON+, and MSON- eye OCTA metrics that might aid in the differentiation of these two conditions are discussed in the Differentiating NMOSD from MS section of this review.

Reports on the OCTA changes in MOGAD patients with a history of ON (MOGADON+) are sparse, with a single study finding a reduction in peripapillary and parafoveal VAD in MOGADON+ eyes compared to a MOGADON- cohort (93).

In the undifferentiated patient, identifying a prior history of optic neuritis that may have not been identified during the acute episode can aid clinicians in focusing their differential on neurologic disease. OCTA shows promise in clarifying which patients have had optic neuritis in the past. This is particularly salient now, as the 2024 revised McDonald criteria presented at the recent 2025 European Committee for Treatment and Research in Multiple Sclerosis (ECTRIMS) Winter School conference includes the optic nerve as a unique compartment in the diagnosis of MS (108). Finally, OCT has been used to identify subclinical optic nerve involvement in clinically isolated syndrome (CIS), which is associated with greater disease burden (109). OCTA studies have yet to examine retinal vascular changes in the setting of subclinical optic nerve changes, and this area warrants investigation.

### 3.5 Alzheimer disease

Alzheimer disease (AD) represents the most common form of dementia, with an estimated 55 million individuals living with the condition (18). Its prevalence is only expected to increase with the aging of the population and it is projected that AD care will cost nearly $1 trillion by 2050 (19). Its pathogenesis is frustratingly unclear, though it has been characterized to be a result of the accumulation of misfolded amyloid-beta and tau proteins on neuropathology (18). There is thought to be a genetic contribution as well and several risk factors such as smoking, traumatic brain injury, and cardiovascular diseases have also been implicated in the development of this complex condition (110). Vascular pathology is exceedingly common in the condition, with up to 85–95% of patients demonstrating substantial amyloid-beta deposition and other abnormalities in the vasculature of the brain (23).

It may take nearly 2–3 decades after the initiation of the disease process for clinical symptoms to appear, making early detection, and measurement and monitoring of potential future treatments, critical in preventing irreversible cognitive decline and preserving quality of life (17). Several biomarkers have been proposed as potential tools for improving the early detection of AD, with preclinical AD broadly defined as those who are cognitively healthy with at least one positive biomarker (18). Currently, reduction in CSF amyloid-beta-42 and visualization of cerebral amyloidosis on positron emission tomography (PET) are the most commonly used biomarkers in the preclinical classification of AD (30). However, CSF testing is invasive with inherent risks and PET imaging is commonly cost-prohibitive for many patients (22). Much work has been done in recent years to characterize changes in the exquisitely small retinal microvasculature in both preclinical and diagnosed AD patients on OCTA, which has the benefit of being completely non-invasive and substantially less costly than classical techniques. Below, we summarize correlations between OCTA metrics and systemic features of AD.

#### 3.5.1 Correlation with cognitive testing

Several different clinical tests are available to assess cognitive function, including the Mini Mental State Exam (MMSE) and Montreal Cognitive Assessment (MoCA). Decreases in macular VAD and VLD as well as RPCP VAD and VLD have been correlated to worse MMSE and MoCA scores in AD and AD-spectrum mild cognitive impairment (MCI) in several studies (111–120). Decreases in FAZ area have also been correlated with worse scores on MMSE and MoCA in AD (111). FAZ circularity index has also been shown to positively correlate with MMSE scores in AD (121). However, several studies have failed to identify a correlation between OCTA parameters and MMSE or MoCA scores in AD and MCI (122–125). Additionally, it is important to note that several of the studies that found meaningful relationships grouped patients with AD with healthy controls or with those with other types of dementia such as vascular dementia and posterior cortical atrophy, limiting our ability to draw conclusions about the relationship between OCTA parameters and cognitive measures in AD specifically (111–114, 116–122, 125), while another failed to correct for multiple comparisons (115).

Longitudinally, a large study failed to find correlations between SCP, ICP, or DCP VAD or FAZ area and global cognitive function based on a battery of 10 neurocognitive tests, change in global cognitive function over time, or incident MCI or dementia following adjustment for confounding variables (126). A different study in patients diagnosed with amnestic MCI identified that higher RPCP VAD reduction was significantly associated with reductions in MMSE score over time (127). Interestingly, this study found that only amnestic MCI non-converters to AD showed significant longitudinal DCP VAD reduction while converters to AD did not (127), suggesting the largest depletion of macular vessels might occur early in the disease course prior to clinical diagnosis, with continued, slower depletion with continued disease. VLD of the full retinal network and SCP has been shown to be reduced more in those with diagnosed AD compared to those with MCI (123, 128). AD patients have similarly exhibited a reduction in SCP and DCP VAD compared to individuals with MCI (129). However, other studies have failed to identify any significant differences between MCI and AD with respect to VAD, VLD, or FD (125, 130).

With respect to other measures of cognition, SCP VLD was positively correlated with working memory performance in those with Alzheimer-spectrum MCI (131), and SCP VAD and VLD were positively correlated with visuospatial and executive functioning (116), suggesting that OCTA parameters may exhibit changes reflective of alterations in cognition.

#### 3.5.2 Correlation with neuroimaging

Decreases in SCP VAD have been correlated with increases in MRI-based brain atrophy scores (113, 132). Specifically, SCP VAD has exhibited significant positive associations with whole hippocampus volume and hippocampal subfield volumes on MRI in cognitively impaired patients on the spectrum of AD (133, 134). In early onset AD patients, SCP, ICP, and DCP VLD all linearly correlate with parietal lobe volume (135). Functionally, reduction in macular SCP VLD has been associated with declines in the connectivity of the cortical visual system in those with AD spectrum cognitive impairment (136).

Cognitively healthy individuals with amyloid-beta positivity identified on PET had higher VAD than amyloid-beta negative patients in all macular regions and around the optic nerve head with no difference in FAZ area in one study (137), but reduced foveal SCP and DCP VAD and larger FAZ area in another study (119). At the same time, amyloid-beta positive patients with AD had no difference in SCP VAD or VLD relative to amyloid-beta negative patients with AD, and SCP VAD and VLD were lower in a mixed group of patients with AD, patients with MCI, and cognitively healthy individuals in a different study, suggesting that changes on OCTA as a result of amyloid-beta pathology might vary by stage of AD (121). Significant differences in study design and cohorts might explain the opposing conclusions reached by the articles. Regarding the FAZ, a separate study found increased FAZ area in individuals with preclinical AD compared to those without at 3-year follow-up (138).

SCP VAD has been found to negatively correlate with Fazekas scale, which measures the severity of MRI-detected brain white matter hyperintensities (WMH) (113, 139, 140), though this finding has not been universal (122, 134). SCP, DCP, and full retinal VAD have also been significantly inversely associated with WMH volumes on MRI (119).

In a cohort consisting of patients with subcortical vascular and amyloid-positive Alzheimer-disease-related cognitive impairment, RPCP VAD has been negatively correlated with brain vascular health as quantified by the cerebral small vessel disease score, which incorporates volume of white matter hyperintensities, number of lacunes, and microbleeds on brain MRI (141). Lower SCP VLD has also been associated with worse MRI measures of cerebrovascular reactivity and reduced perfusion of the middle cerebral artery perfusion territory in Latinx patients (118). OCTA parameters have not exhibited relationships with neuroimaging universally, as some studies have failed to identify any meaningful relationships (124, 125, 142, 143). The correlation of OCTA metrics with neuroimaging in AD continues to be an active area of research, with potential to provide important information on the brain’s underlying vascular health.

#### 3.5.3 Correlation with serum and CSF biomarkers

Initial studies have not identified correlations between OCTA parameters and CSF amyloid-beta or tau protein levels (113, 116, 139, 140, 144, 145). One study identified a negative correlation between DCP VAD and p-tau181; however, this study was conducted in those with neovascular age-related macular degeneration and cataracts, which are known to impact OCTA parameters (146). Additionally, reduced CC VLD has been correlated with increased serum levels of amyloid-beta-42, amyloid-beta-42/40 ratio, and p-tau 181 levels in patients with early onset AD (135).

The CSF amyloid-beta-42-to-total-tau ratio has been found to negatively correlate with FD, however (144). Those with pathologic ratios indicative of presymptomatic AD have also exhibited reduced VAD, VLD, and FD in the SCP, increased FAZ area, and choriocapillaris flow deficits (147). FAZ area has also been reported to be larger in cognitively normal individuals with either PET or CSF amyloid-beta positivity (148). Significant differences have been identified in amyloid-beta positive patients identified on PET, which are discussed above in the Correlation with Neuroimaging section. Overall OCTA shows promise in reflecting changes in systemic AD biomarkers, particularly in those with preclinical disease.

#### 3.5.4 Genotypic differences

Risk of AD is estimated to be 60–80% dependent on genetic factors, and the apolipoprotein E (APOE) alleles exhibit the strongest association with AD development (149). APOE is a glycoprotein that plays a critical role in cholesterol and lipid metabolism (150). Three variants of the ApoE gene exist with ε3 being most common (78%), followed by ε4 (14%) and ε2 (8%) (150). ApoE-ε4 is known to increase risk for AD up to 15-fold while also causing high LDL, high triglycerides, and low HDL and an increased risk of cardiovascular disease, while ε3 is neutral and ε2 is protective (150). In addition, APOE-ε4 increases amyloid-beta accumulation in the cerebral vasculature (151, 152). Several significant relationships have been identified between APOE status and OCTA metrics.

APOE-ε4 allele carriers have exhibited reduced macular and peripapillary VAD and VLD across the spectrum of AD (113, 153, 154). Specifically, reduction in SCP VAD and VLD as well as CC VLD have been observed in multiple studies of APOE-ε4 allele carriers (135, 155). One of these studies identified reduced SCP VAD in their entire cohort of APOE-ε4 allele carriers, but interestingly also found a reduction in the DCP in those with cognitive impairment (155). This suggests that changes in the SCP might precede DCP changes that might be more likely to be observed with disease progression in AD. The APOE-ε4 allele has been closely linked to vascular changes in AD, exhibiting a greater influence on retinal microvascular metrics (SCP B = −1.638, DCP B = −0.772) than did cognitive scores (SCP B = −1.397, DCP B = −0.674) in one relative importance analysis using partial regression coefficients (155). In fact, APOE-ε4 status may explain up to 18% of variance in VAD and up to 14% of variance in VLD (143) in APOE-ε4 carriers.

Given the complex interplay of genotype and family history (FH) in the onset of AD, some researchers have also controlled for FH into their analyses. One such study identified lower VAD in a FH + and APOE-ε4 + cohort compared to FH + and APOE-ε4- individuals (156). Another study identified a smaller FAZ area in a FH + and APOE-ε4 + cohort compared to FH + and APOE-ε4- cohort, though this study was confounded by including those with hard drusen, which are known to affect OCTA parameters (157). Periarteriolar and perivenular capillary free zone width, which approximate how far oxygen must diffuse to tissues surrounding the ophthalmic posterior segment arterioles and capillaries, have differed between those with at least one APOE-ε4 allele and a positive first-degree FH of AD (high-risk) compared to low-risk individuals with neither of these characteristics (124). Overall, these findings further support the concept that APOE-ε4 status may be a key driver behind the microvascular changes observed in AD. Despite the majority of studies identifying significant differences in OCTA parameters based on APOE-ε4 genotype, these findings have not been universal (140, 158).

Beyond the APOE-ε4 allele, one study explored the OCTA differences in those with autosomal dominant AD-causing mutations (159). Researchers divided those with PSEN1 or APP mutation into early stage (ES) and late stage (LS) and found that ES carriers had significantly greater average OCTA capillary signal intensity than both controls and LS patients (159). This finding supports the hypothesis that vascular changes may be a hallmark of preclinical AD.

#### 3.5.5 Differentiating AD from other forms of dementia

Incorporating OCTA parameters into a multi-regression framework to discriminate MCI from AD did not improve the model’s performance (160), suggesting OCTA may not add value in differentiating these conditions. Further, it appears that no difference exists in the OCTA parameters of those with early-onset AD compared to late-onset AD (161). At the same time, OCTA has shown promise in helping to differentiate AD from other dementia subtypes (162). Specifically, those with subcortical vascular cognitive impairment (SVCI) have exhibited reduced VAD in the superior and temporal quadrants of the RPCP compared to patients with amyloid-beta positive AD-related cognitive impairment (141). In addition, a model combining MoCA results, retinal thickness, and ICP VAD and VLD has shown the best ability to discriminate between AD and posterior cortical atrophy (AUC = 0.944), which typically presents with prominent visual dysfunction with preservation of executive functioning, making early diagnosis particularly difficult (121).

#### 3.5.6 Conclusion

Across the spectrum of AD, correlations between retinal microvascular perfusion and disease stage, cognitive testing scores, and CSF (but not serum) biomarkers, have been widely observed, with decreases in perfusion typically corresponding to worse disease. The underlying pathophysiology of this vascular loss might be specific to AD. Vascular mural cells (VMCs) express APOE, and APOE-ε4 expression in VMCs has been associated with a reduction in arteriole blood flow and an impaired ability to support endothelial cell function (151). Recent research in diabetes has suggested that loss and dysfunction of retinal VMCs may be a key driver in the widely observed reduction in retinal microvasculature measured on OCTA in that disease (163). Thus, VMC dysfunction secondary to APOE-ε4 expression might drive the reduction in retinal vasculature observed in AD. The specific subpopulations of VMCs in the retina, their functions, and their localization is a matter of ongoing research, and it remains possible that certain populations of VMCs play an outsize role in such a process. Further study of the relationship between APOE-ε4 expression in VMCs and vascular changes is warranted to investigate these possibilities. Amyloid-beta might also independently contribute to the vascular pathogenesis of AD, as it acts as a capillary constrictor (164), which might provide another explanation for the observed reductions in microvasculature. It is possible that identification of the reported microvascular attenuation in AD and its precursors might help to initiate lifestyle changes and other interventions at disease inception to prevent the clinical onset of symptoms, though much more research is needed.

### 3.6 Parkinson disease

Parkinson disease (PD) is the 2nd most common neurodegenerative disease, commonly manifesting with motor dysfunction and cognitive impairment (165). Nearly 80% of patients will present with visual symptoms, and histopathologic analyses have revealed decreased dopamine as well as phosphorylated alpha-synuclein deposits in the retina, which are widely accepted markers of PD (166, 167). In addition to neuronal degeneration, vascular degeneration is thought to play a role in PD’s pathogenesis as well (167). With PD most commonly diagnosed clinically, there is a growing need for objective biomarkers to improve diagnosis of the condition (167). While dopamine transporter (DaT) scans have shown clinical utility in altering the diagnosis and management of suspected PD patients, the technology is quite expensive and not available in all healthcare systems (168). OCTA shows promise in filling this gap, as summarized below.

#### 3.6.1 Correlation with disease stage

OCTA changes in PD have shown promise in differentiating patients in different stages of the condition. Two common scores for staging PD are the Movement Disorder Society-Unified Parkinson’s Disease Rating Scale (MDS-UPDRS) and the Hoehn-Yahr (H-Y) stages. DCP macular and RPCP VAD have been shown to be significantly lower in patients with moderate-stage compared to early-stage PD based on MDS-UPDRS (169). Reduced FAZ area has been correlated with worsening H-Y stages of disease, as has an increase in the number of SCP macular subfields with reduced VLD (165). SCP VAD has similarly been negatively correlated with H-Y and MDS-UPDRS staging (170). Interestingly, the rate of change in SCP VAD and VLD has been shown to be faster in H-Y stages 3–4 compared to H-Y stages 1–2.5, suggesting that the microvascular rarefaction broadly observed in PD may only accelerate with disease progression (171). Other studies have failed to correlate OCTA parameters with PD stage (172–174).

Studies investigating relationships between the RPCP and disease stage have been less consistent with one group observing a negative correlation between RPCP VAD and disease stage while another failed to identify any meaningful relationship (166, 167).

There is also emerging evidence that OCTA may be able to capture preclinical changes in PD. Patients with idiopathic rapid eye movement sleep behavior disorder, the strongest marker in the diagnosis of prodromal PD, have been found to have elevated DCP VAD compared to both healthy controls and patients with PD (175). Additionally, PD onset is often asymmetric, and the eyes ipsilateral to motor-symptom-onset have exhibited reduced SCP and DCP VAD (176). Further, PD patients with MCI have been found to exhibit an increased FAZ area and reduced FAZ circularity compared to PD patients without MCI (177). Such microvascular changes might eventually assist in differentiating PD from other disorders of ocular motility after future research and might accelerate relevant diagnostic testing and treatment initiation. Of note, due to the common motor symptoms observed in PD, such as resting tremor, it will be important for future studies to consider and control for motion artifacts in OCTA imaging (178).

#### 3.6.2 Identifying symptom presence and predicting symptom progression

In addition to differentiating PD by stage, OCTA has also shown potential in differentiating patients based on symptom burden and predicting longitudinal symptom progression. Including OCTA metrics in a statistical model improved the model’s ability to predict cognitive symptom progression at one-and-half years (169). However, the incorporation of OCTA metrics did not improve a different statistical model’s ability to predict motor symptom progression at one-and-half years (169). Another study found no difference in SCP VAD between patients with PD and those with essential tremor (179), further supporting the notion that OCTA may be incapable of predicting motor symptom progression. With respect to autonomic symptoms, PD patients with orthostatic hypotension (OH) exhibit reduced DCP VAD compared to those without OH (180). DCP VAD has also been negatively correlated with changes in blood pressure during the head-up tilt test, suggesting that DCP VAD may be able to shed light on the degree of autonomic dysfunction in those with PD and OH (180). These results indicate that OCTA’s ability to predict symptom progression and differentiate patients based on symptom presence, particularly in the cognitive and cardiovascular domains, in PD and other neurologic disease should be investigated further.

#### 3.6.3 Conclusion

As with many neurodegenerative diseases, there is emerging evidence that PD pathophysiology has an underlying vascular component. Pericyte dysfunction has been proposed as a potential cause of the vascular changes observed in the substantia nigra, the brain center responsible for the predominant motor symptoms observed in PD (181). PD mouse models have exhibited an initial angiogenic period in early disease followed by extensive microvascular pruning with disease progression (181). Existing human ophthalmic posterior segment OCTA research parallels these findings, with an increase in DCP VAD observed in prodromal PD and an accelerating microvascular depletion with worsening disease. Further study is needed to understand the exact cause of pericyte dysfunction that might initiate this process, though a leading theory suggests that exposure to alpha-synuclein activates pericytes and ultimately causes abnormal functioning (181). OCTA studies correlating parameters with quantitative alpha-synuclein assays, CSF markers of angiogenesis, and quantitative imaging studies of the substantia nigra pars compacta might help to establish OCTA parameters as biomarkers capable of providing critical information on the microvasculature of PD patients.

## 4 Overall discussion, limitations, and conclusions

Broadly, across MS, NMOSD, MOGAD, AD, and PD, ophthalmic posterior segment OCTA has shown promise as a potential future biomarker for disability, cognitive functioning, structural and functional changes on neuroimaging, alterations in accepted systemic markers of disease, symptom burden, disease detection, and longitudinal symptom progression. Further, OCTA has shown significant potential in aiding in the diagnosis of preclinical disease, particularly in AD and PD. Early detection is of the utmost importance in these irreversible neurologic diseases, as well as monitoring for disease progression and response to treatment. OCTA’s unique non-invasive nature and relative affordability might provide it with significant advantages over many classical approaches.

Few reviews similar to this one seeking to relate quantitative ophthalmic OCTA metrics to systemic markers of non-ocular diseases have been performed. Those that have been conducted (4, 5), have, like the current study, found that OCTA metrics may be of clinical relevance in a variety of systemic conditions not previously thought to be linked to the eyes.

Strengths of this study include the broad variety of OCTA metrics and systemic disease markers included. Further, including discussion regarding both the statistically significant and not statistically significant correlations identified in the literature helps provide a balanced view of current findings in this field. Limitations of this review include conducting search queries only in the PubMed database and only including articles in the English language. Reporting bias is a potential confounder, as insignificant results may have been less likely to be published in the literature; however, a reasonable balance of statistically significant and statistically insignificant results was identified in the reported works. Further, many of the studies reported here were conducted at a single time point, making it difficult to draw conclusions regarding causality, though several longitudinal studies have been performed. In addition, the effect of aging, which typically results in lower values for quantitative OCTA microvascular metrics, merits scrutiny for possible age differences between subjects and controls.

Nonetheless, the initial results reviewed here suggest the potential utility of OCTA in diagnosis, prognosis, and monitoring in neurologic diseases. Implications of the reported findings include that, if borne out by further study, OCTA might allow for differentiation between diseases that frequently mimic each other, like multiple sclerosis, neuromyelitis optic spectrum disorder, and myelin oligodendrocyte antibody associated disease. OCTA might similarly allow Alzheimer disease to be distinguished from other forms of dementia. At the same time, significant research must be undertaken to validate these preliminary findings and determine ideal potential use-cases for OCTA in these conditions and provide further insight into whether these vascular changes are part of disease pathogenesis or the aftermath of neuronal damage. Prospective studies examining the utility of OCTA in the diagnosis of neurologic diseases, especially in populations of disease-suspects, are merited. Additionally, research into the time course of OCTA changes relative to the development of clinically significant disease ought to be carried out. Furthermore, machine learning and artificial intelligence models should be constructed to better delineate the utility of OCTA in providing insights into systemic aspects of neurologic diseases, as has been done with color fundus photography (182, 183).

## Data Availability

The original contributions presented in the study are included in the article/supplementary material; further inquiries can be directed to the corresponding author.
